# Heterosexual oral and anal sex in Kinshasa (D.R.Congo): Data from OKAPI prospective cohort

**DOI:** 10.1371/journal.pone.0210398

**Published:** 2019-01-16

**Authors:** Silvia Carlos, Cristina López-del Burgo, Adolphe Ndarabu, Alfonso Osorio, Anaïs Rico-Campà, Gabriel Reina, Eduardo Burgueño, Jokin de Irala

**Affiliations:** 1 Preventive Medicine and Public Health Department, University of Navarra, Pamplona, Spain; 2 IdiSNA, Navarra Institute for Health Research, Pamplona, Spain; 3 Institute for Culture and Society (ICS), Education of Affectivity and Human Sexuality, University of Navarra, Pamplona, Spain; 4 Monkole Hospital, Kinshasa, Democratic Republic of Congo; 5 School of Education and Psychology, University of Navarra, Pamplona, Spain; 6 Biomedical Research Centre Network on Obesity and Nutrition (CIBERobn), Institute of Health Carlos III, Madrid, Spain; 7 Microbiology Service, Clínica Universidad de Navarra, Pamplona, Spain; 8 School of Medicine, University of Mwene-Ditu, Mwene-Ditu, Democratic Republic of the Congo; Greenebaum Cancer Center, Institute of Human Virology, University of Maryland School of Medicine, UNITED STATES

## Abstract

**Background:**

Sexually transmitted infections can be spread through oral and anal heterosexual sex. There are few data on these practices in Sub-Saharan Africa. We analyzed the prevalence of heterosexual oral and anal sex among HIV Voluntary Counseling and Testing (VCT) attendees in Kinshasa and the associated sociodemographics, perceptions and behavioral factors.

**Methods:**

OKAPI (Observational Kinshasa AIDS Prevention Initiative) prospective cohort study. It evaluates the VCT impact on HIV-related knowledge and behaviors at 6 and 12-month follow-up. Since April 2016 until April 2018, 797 persons aged 15–59 years were HIV tested and replied to a baseline interview, including information about anal and oral sex. Descriptive, bi- and multivariate analyses were performed using baseline data.

**Results:**

Among 718 sexually active participants reporting heterosexual sex, 59% had had oral sex, 22% anal sex and 18% both practices. Among participants reporting “not” having had sex, 6% reported oral sex, 3% anal sex and 1% both. Oral sex was associated with a daily use of the Internet/mobile phone, perceiving low community HIV risk, reporting HIV-related behaviors (multiple partners, inconsistent condom use, anal, paid and forced sex) and having been pregnant. Being married-monogamous was inversely associated with oral sex. Anal sex was directly associated with having other risk sexual behaviors.

**Conclusions:**

Oral and anal sex were common among people reporting heterosexual sex in Kinshasa. Perceiving a low community HIV risk and having other sexual risk behaviors are associated with these practices, which are commonly not considered as risky despite their strong association with HIV/STIs. They need to be considered when designing preventive strategies in Kinshasa.

## Introduction

Although the incidence of HIV has declined in the last decade, in 2016 there were still 1.8 million people newly infected, and about 64% of new infections took place in Sub-Saharan Africa (SSA), where more than half of new infections are among women aged 15–24 years [1,2]. In this region, sexual transmission is the main infection route, mostly through heterosexual contact [3].

Most sexual prevention strategies are focused on biomedical approaches (such as condom or Pre-Exposure Prophylaxis use) and some behavioral ones (as the reduction in multiple sexual partnerships or the avoidance of early sex) [4–6]. However, official preventive recommendations focused on HIV and other Sexually Transmitted Infections (STIs) usually do not include messages about oral or anal sexual practices. Neither are there data on these specific behaviors for many SSA countries [7–21].

However, these extravaginal sexual practices carry a high risk, although people do not perceive it. Many different symptomatic and asymptomatic STIs can be spread through oral or anal sex, including HIV, Human Papillomavirus, Herpes Simplex Virus, Hepatitis B Virus, *Chlamydia trachomatis*, *Neisseria gonorrhoeae*, *Treponema pallidum* or *Trichomonas vaginalis* [22–29]. Other genital microorganisms have also been detected in the oral cavity, such as *Ureaplasma urealyticum*, *U*.*parvum*, *Mycoplasma hominis*, or *Mycoplasma genitalium* [30,31]. Furthermore, other agents transmitted through feces need to be considered, such as Hepatitis A Virus, *Shigella*, *Salmonella*, *Campylobacter or Entamoeba histolytica*, among others. It is now well known that, apart from the sexual and reproductive consequences of these infections [32], different types of cancers can also be developed (oropharyngeal, anal, cervical, vulvar, vaginal or penile cancers). In spite of all these risks, people (and mainly adolescents) have oral or anal sex believing they can avoid both pregnancy and STIs. They even consider that these practices are not “having sex” [33–37].

According to recent data, oral and anal sex are highly prevalent. In high-income countries, national surveys and different studies in the general population have shown a prevalence of people reporting heterosexual oral sex higher than 50% [38–41] and a prevalence around 30% of people reporting heterosexual anal sex [39,41]. Furthermore, there has been an increase in the prevalence of both oral and anal sex (particularly among 16- to 24-year-olds) [42]. In SSA, information on these practices is insufficient among the general population and regarding oral sex. The reported prevalence of oral sex comes mainly from young people and ranges from 8% to 52% [43–45], although some particular studies have shown even higher frequencies [46]. Regarding anal sex, figures range from 4% to 57% [8,16,17,43,44,47–50], with most of the information coming from South African studies.

In the Democratic Republic of Congo (DRC) there are no research data on oral and anal sexual practices, neither in the national Demographic and Health Survey [51]. Although the DRC is not among the SSA countries with the highest HIV prevalences or incidences and that there has been a decline in the estimated people affected by HIV/AIDS, in 2017 390,000 people were estimated to be living with HIV. Among them 59% know their status and 93% of them were on antiretroviral treatment. Therefore, the country is far from reaching UNAIDS 90-90-90 goals for 2020 (90% of people living with HIV knowing their HIV status; 90% of them receiving antiretroviral therapy and 90% of people on treatment having viral suppression). At the same time, the DRC is among the countries with the highest prevalence of girls reporting sex before 15 years [52]. Therefore, great preventive efforts should be made to avoid new HIV infections and other STIs, especially among youth.

In this study we aim to describe the prevalence of oral and anal sex among participants attending Voluntary Counseling and Testing (VCT) in Kinshasa reporting heterosexual sex. Also, we intend to analyze the sociodemographics, perceptions and behavioral factors associated with these practices.

## Methods

### Study design and participants

Since April 2016 until April 2018 all people aged 15–59 years attending HIV VCT at a reference hospital in Kinshasa were offered to participate in the OKAPI (Observational Kinshasa AIDS Prevention Initiative) prospective cohort study. All those accepting to participate were included in this cohort. It evaluates the impact of the HIV VCT on changes in HIV knowledge and sexual behaviors 6 and 12-months after VCT. People that had received a previous HIV positive test as well as pregnant women were excluded.

### Data collection

Face-to face interviews were carried out at baseline and at 6- and 12-month follow-up. Male and female interviewers were available. Through a pen and paper questionnaire they collected data about the participants´ sociodemographics, HIV-related knowledge and beliefs, attitudes, behaviors and exposure to HIV-related information. Information about oral sex (fellation and cunnilingus) and anal sex was collected for both male and female participants (insertive anal sex only among men). The possible answers were: `never´, `rarely´, `often´ and `I don´t want to answer´. Two new variables were created, `having ever had oral sex´ or `having ever had anal sex´. In both cases we collapsed the categories `rarely´ and `often´ into the category `yes´ and `never´ and `I don´t want to answer´ into `no´.

### Laboratory analyses

A blood sample was collected from each participant. Consistent with the national protocol in Congo-Kinshasa for HIV diagnosis, rapid diagnostic tests were used: first, Determine HIV-1/2 test and if positive, DoubleCheckGold and Unigold rapid immunoassays.

### Statistical analyses

The main outcomes were having ever had oral sex or anal sex. Sociodemographic and behavioral independent variables were included in the analyses.

We first carried out a descriptive analysis to evaluate the baseline characteristics of study participants. Secondly, we carried out univariate logistic regressions to evaluate the characteristics of participants crudely associated with reporting and not reporting oral and anal sexual practices as well as age-adjusted models. Finally, to evaluate the association between participants´ socio-demographics, knowledge/perceptions and behaviors and anal/oral sex, multivariate logistic regression models including all significant variables of the univariate regressions were conducted. An additional multivariate analysis was carried out to study the association between anal/oral sex and an incident HIV positive test or an STI diagnosis.

All p-values<0.05 were considered statistically significant. Analyses were performed using STATA version 12.1 (StataCorp, College Station, TX, USA).

### Ethical issues

Ethical approval from the Research Ethics Committees of the two centers involved was obtained. A written informed consent was collected from each participant or from their parents/guardians in case they were minors.

## Results

### Participants´ characteristics

During the study period, 797 participants (mean age: 30; SD: 9.4) were HIV tested and replied to the baseline interview.

At baseline, 58% of participants were women and 32% were aged 15–24 years, most of them women (77%). The majority of the participants reported a middle economic level (76%) and university studies (67%), with 10% of men and 16% of women being students at the time the study took place. Most participants were single (83% of men and 92% of women). Regarding their religion, 95% of participants reported a weekly or daily religious practice.

In general, participants requested VCT individually (84%) although some attended it with their partner (16%). Most people reported previous HIV screenings (64% at least one previous test and 20% two or more). However, only 3% of the participants perceived themselves as having a high HIV risk (1% of young aged 15–24 and 3% of adults) and 10% affirmed having ever had an STI diagnosis. When the HIV test was carried out, 3% of participants got a new HIV positive diagnosis at baseline.

Regarding HIV knowledge, although 75% reported talking about HIV/AIDS sometimes or often, misconceptions related to HIV transmission were highly prevalent, with 38% of participants thinking that HIV is transmitted through witchcraft or God´s punishment, 24% through social kiss and 17% through mosquito bites.

### Sexual behaviors

**Fig 1:**
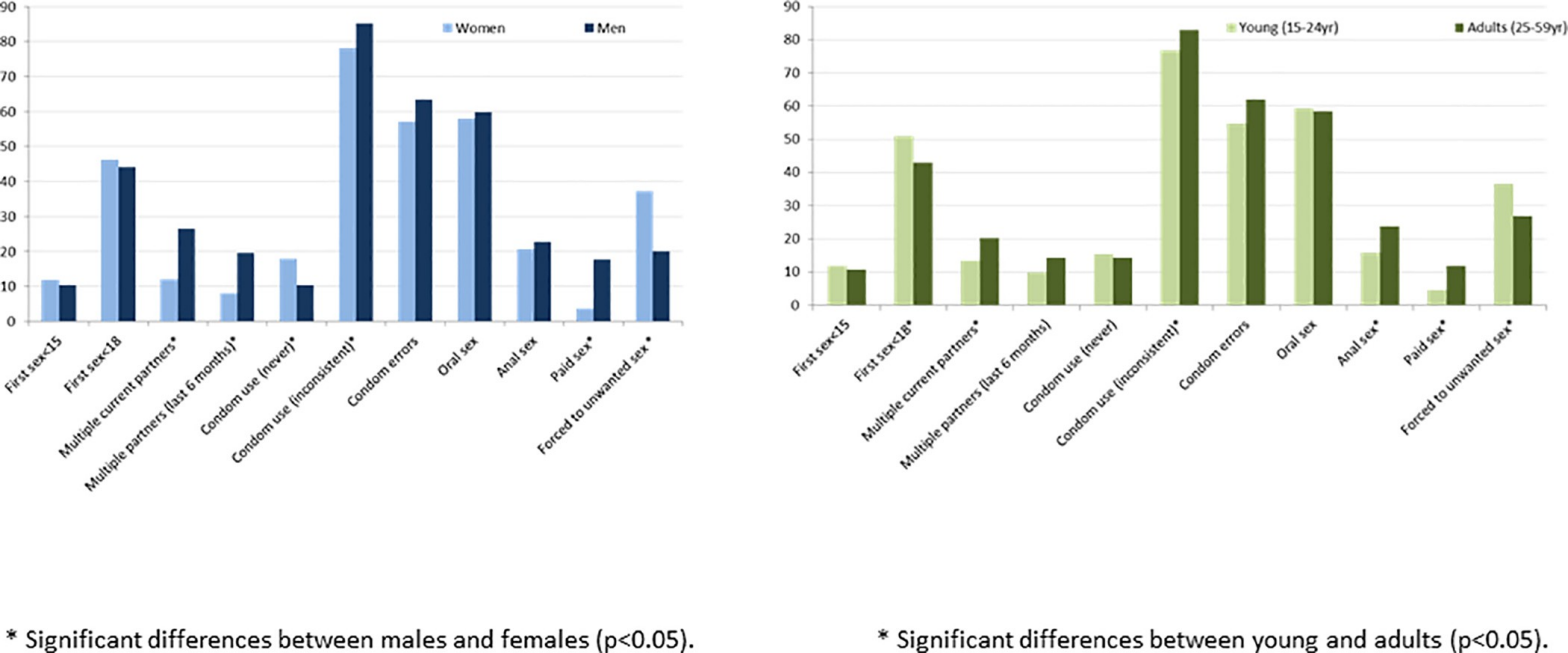
Sexual behaviors among the sexually active OKAPI cohort participants (N = 718).

With regards to sexual behaviors we found that most participants have had sexual relationships (80% of young and 97% of adults, p<0.001). Fig 1 shows the main HIV-related risk behaviors among sexually active participants reporting heterosexual sex: 11% had had sex before 15 years, 34% at 15–17 years and 45% before 18 years; multiple sexual partnerships were significantly more frequent among men than women, both concerning concurrent partners at study time (26% vs. 12%, p<0.001) and serial partners in the previous six months (20% vs. 8%, p<0.001). Regarding condom use, 93% had ever used condoms, but only 3% had used them consistently (no significant differences existed between women and men, but consistent use was significantly more frequent among young than adults). Among condom users, 60% reported at least one condom use error (late application, early removal, breakage, slippage or leakage), with no significant differences between age and age subgroups. The main reason for condom use was to reduce the probability of pregnancy (67%) and only 23% used them for HIV or STI prevention.

Among sexually active participants reporting heterosexual sex, 59% reported having ever had oral sex (55% fellatio and 55% cunnilingus among women, and 58% and 43% respectively, among men). Regarding anal sex, 19% of women reported receptive anal sex and 23% of men reported insertive anal practices. Nearly one out of five participants had ever practiced both oral and anal sex (18%). Among participants reporting heterosexual oral sex, only 2% were consistent condom users and 8% had never used condoms. For participants reporting anal sex, the percentages were 1% and 6%, respectively.

Among participants reporting “not” having had sex at baseline, we found that 6% had had oral sex, 3% anal sex and 1% both oral and anal sex.

### Factors associated with reporting heterosexual oral and anal sex

When factors associated with oral sex were analyzed (Table 1), we found that reporting oral sex was positively associated with having a daily use of the Internet and the mobile phone (OR = 1.6; 95% CI: 1.1–2.3 and OR = 3.9; 95% CI: 1.9–8.0, respectively), perceiving low HIV risk in the community (OR = 2.4; 95% CI: 1.5–3.9), having ever been pregnant (OR = 2.3; 95% CI: 1.4–3.8) and having HIV-related sexual risk behaviors, such as multiple sexual partners (OR = 2.3; 95% CI: 1.4–3.9), inconsistent condom use (OR = 2.8; 95% CI: 1.7–4.5), anal sex (OR = 3.6; 95% CI: 2.2–5.9), paid sex (OR = 2.1; 95% CI: 1.0–4.3) and forced sex (OR = 1.9; 95% CI: 1.3–2.9). On the contrary, an inverse association with oral sex was found for being in a monogamous marriage (OR = 0.5; 95% CI: 0.3–1.0).

**Table 1:** Factors associated with oral and anal sex among the sexually active OKAPI cohort participants (N = 718).

			ORAL SEX					ANAL SEX		
	No	Yes	Crude OR	Age-adj OR	aOR*	No	Yes	Crude OR	Age-adj OR	aOR**
	(N = 296)	(N = 422)	(95%CI)	(95%CI)	(95%CI)	(N = 563)	(N = 155)	(95%CI)	(95%CI)	(95%CI)
	(%)	(%)				(%)	(%)			
Sex: women vs. men	57.8	55.9	0.9 (0.7–1.2)	0.8 (0.6–1.1)	0.8 (0.5–1.2)	57.4	54.2	0.9 (0.6–1.3)	1.0 (0.7–1.4)	1.0 (0.5–1.7)
Age (yrs.): 25–59 vs. 15–24	72.3	71.6	1.0 (0.7–1.3)	—	0.7 (0.5–1.1)	69.8	79.3	1.7 (1.1–2.5)	—	1.7 (1.0–2.7)
Married-monogamous: `Yes´ vs. other	13.8	6.2	0.4 (0.2–0.7)	0.5 (0.3–0.8)	0.5 (0.3–1.0)	9.1	10.3	1.2 (0.7–2.2)	1.1 (0.6–2.0)	—
Media use										
The internet (daily): `Yes´ vs. `no´	39.5	53.1	1.7 (1.3–2.3)	1.7 (1.2–2.3)	1.6 (1.1–2.3)	47.1	49.0	1.1 (0.8–1.5)	1.2 (0.8–1.7)	—
Mobile phone (daily): `Yes´ vs. `no´	87.8	96.2	3.5 (1.9–6.5)	3.4 (1.9–6.3)	3.9 (1.9–8.0)	92.9	92.3	0.9 (0.5–1.8)	0.9 (0.5–1.8)	—
Perceive low community HIV risk: `Yes´ vs. `no´	82.1	89.6	1.9 (1.2–2.9)	1.9 (1.2–2.9)	2.4 (1.5–3.9)	86.5	86.4	1.0 (0.6–1.7)	1.0 (0.6–1.7)	—
Alcohol consumption (daily): `Yes´vs. `no´			2.7 (1.3–5.5)	2.8 (1.4–5.8)	1.9 (0.8–4.3)			2.5 (1.3–4.7)	2.4 (1.3–4.5)	1.6 (0.8–3.1)
Age at first sex (yrs). `Yes´ vs. `no´	42.6	47.2	0.9 (0.9–0.9)	0.9 (0.9–1.0)	0.9 (0.9–1.0)	41.6	58.7	0.9 (0.9–1.0)	0.9 (0.9–1.0)	0.9 (0.9–1.0)
Ever pregnant (women). `Yes´ vs. `no´	42.7	61.0	1.6 (1.1–2.2)	1.6 (1.1–2.2)	2.3 (1.4–3.8)	48.9	70.2	1.6 (1.1–2.3)	1.6 (1.1–2.3)	1.5 (0.9–2.7)
Multiple concurrent sexual partners. `Yes´ vs. `no´	9.8	24.2	2.9 (1.9–4.6)	3.0 (1.9–4.7)	2.3 (1.4–3.9)	14.9	30.3	2.5 (1.6–3.7)	2.4 (1.6–3.7)	1.6 (1.0–2.5)
Condom use: inconsistent vs. never	68.9	89.8	4.2 (2.7–6.5)	4.4 (2.9–6.9)	2.8 (1.7–4.5)	78.0	92.9	3.9 (1.9–7.8)	3.8 (1.9–7.7)	2.1 (1.0–4.5)
Type of sex										
Anal sex. `Yes´ vs. `no´	8.8	30.6	4.6 (2.9–7.2)	4.7 (3.0–7.5)	3.6 (2.2–5.9)	NA	NA	NA	NA	NA
Oral sex. `Yes´ vs. `no´	NA	NA	NA	NA	NA	52.0	83.2	4.6 (2.9–7.2)	4.8 (3.0–7.6)	3.2 (2.0–5.1)
Paid sex. `Yes´ vs. `no´	4.0	13.7	3.8 (2.0–7.2)	4.1 (2.1–7.8)	2.1 (1.0–4.3)	6.6	21.3	3.8 (2.3–6.4)	3.7 (2.2–6.2)	2.5 (1.4–4.4)
Ever partner-forced to unwanted sex.`Yes´vs. `no´	21.3	35.5	2.0 (1.4–2.9)	2.0 (1.4–2.8)	1.9 (1.3–2.9)	26.8	40.0	1.8 (1.2–2.6)	1.9 (1.3–2.7)	1.5 (1.0–2.2)

*Logistic regression model adjusted for sex, age and the significant variables in the crude model: married-monogamous, use of the Internet, mobile phone use, perception of community risk, alcohol consumption, age at first sex, pregnancy, multiple partners, condom use, anal sex, paid sex and forced sex.

**Logistic regression model including the following variables: sex, age, alcohol consumption, early sex, pregnancy, multiple partners, condom use, oral sex, paid sex and forced sex.

NA: Not applicable.

Anal sex was directly associated with HIV-related sexual risk behaviors (Table 1): multiple sexual partners (OR = 1.6; 95% CI: 1.0–2.5), inconsistent condom use (OR = 2.1; 95% CI: 1.0–4.5), oral sex (OR = 3.2; 95% CI: 2.0–5.1), paid sex (OR = 2.5; 95% CI: 1.4–4.4) and forced sex (OR = 1.5; 95%CI: 1.0–2.2).

### Association between oral and anal sex and HIV and STI diagnosis

We found that 3% of our participants had a new HIV diagnosis and 10% reported having ever been diagnosed an STI but we did not find any significant association between reporting oral or anal sex and an HIV or STI diagnosis, after having adjusted for sex, age, having multiple sexual partners, condom use, forced sex and paid sex.

## Discussion

This is the first analytical study in the DRC showing the prevalence of oral and anal sex among people attending HIV Voluntary Counseling and Testing, and the associated demographic and behavioral factors. Among people attending VCT at a reference hospital in Kinshasa who were sexually active and had heterosexual relationships, 59% reported having ever engaged in oral sex, 22% in anal sex and 18% in both anal and oral sex. Even among participants reporting “not” having ever had sex 6%, 3% and 1% had had oral, anal sex and both practices, respectively. Oral sex was associated with a daily use of the Internet/mobile phone, perceiving low community HIV risk, having ever been pregnant, and reporting HIV-related behaviors. Being married-monogamous was inversely associated with oral sex. Anal sex was directly associated with having other risk sexual behaviors.

Although there are not many studies on these sexual practices in SSA, the available information varies greatly among the different countries and study populations. Data on oral sex from the general heterosexual population is very scarce and mostly limited to young people, with a prevalence ranging from 8 to 52% [43,44]. Concerning data related to people attending VCT in a study carried out in South Africa among heterosexual partners, 78% had ever engaged in oral sex, with 45% having had multiple lifetime oral sex partners and 40% having practiced it before age 21 [53]. In another South African study among people aged 18 years and over visiting HIV VCT centers, 13% had had oral sex with the past three sexual partners [54]. Taking all these data into account we can state that the prevalence of lifetime oral sex in our study population was high, being a risk practice which is not present at all in preventive campaigns or strategies in Kinshasa and therefore should be taken into account.

With regards to anal sex, the highest prevalence in SSA among people not coming from high-risk groups was found in high-school students in Ethiopia among whom 57% reported anal practices [44], but other studies have found lower prevalences [8,10,16,17,43,47–49]. One out of five sexually active people attending VCT in Kinshasa reported anal intercourse. As it was already stated by Halperin in 1999 [55], it has been commonly believed that in SSA heterosexual anal sex practice is rare, but our data and previous evidence show that, although it is probably frequently underreported, the prevalence is remarkably high.

The reasons for practicing oral or anal sex in Africa are: avoidance of vaginal sex, menstruation, fulfillment of male pleasure, but also the prevention of unplanned pregnancy or the reduction of STIs [43,56,57]. However, regarding pregnancy we found that having ever been pregnant was independently associated with oral sex. Contraception has been one of the reasons described in the literature for practicing anal sex but there is no reason described for a higher prevalence of oral sex. A recent study carried out in South Africa among pregnant and postpartum women showed that during pregnancy 44% reported oral sex and 8% anal sex; and 49% and 11%, respectively at postpartum [9]. Teasdale et al. found in a study carried out with pregnant women from Zimbabwe and South Africa, that pregnancy decreased sexual activity and high-risk sex, including anal sex [49].

Regarding the perception that extravaginal practices may reduce the risk of STIs, as we previously said, oral and anal sex are strongly associated with a high risk of different STIs [15,20,58]. Kalichman et al. stated that `*replacing all acts of anal intercourse with vaginal intercourse would reduce the mean risk of HIV acquisition by approximately 24%*´ [15]. If we take into account that the prevalence of STIs is increasing in most countries (including the young group which are particularly susceptible) [7], we can realize that ignoring these practices could imply missing important opportunities for HIV and other STIs prevention [59]. Moreover, omitting messages about these practices could be even misinterpreted as if they were safe [56]. Oral and anal sex are rarely discussed in HIV prevention strategies and there is widespread lack of knowledge about their risks [20,60]. Therefore, there is an urgent need of informing the general population about the risks of oral and anal sexual practices [36]. As an example, the PREPARE (Promoting sexual and reproductive health among adolescents in southern and eastern Africa) intervention carried out in Tanzania did take into account the risks of anal sex and was effective in changing sexual risk behaviors [19,61]. One of the UNAIDS commitments to end AIDS by 2030 is to `ensure by 2020 that 90% of young people have the skills, knowledge and capacity to protect themselves from HIV in order to reduce the number of new HIV infections´ [62]. High-quality education is needed to achieve this goal [63].

With regards to age, we could assume that it would be associated with a higher probability of having had a sexual practice; however, as an effect of generational aspects, and looking at the actual tendencies, young people have today a higher probability of engaging in these extravaginal practices which frequency has increased in the last decades [40]. We did not find a significant association for oral sex but we did find an association between anal sex and an older age (25–59 years) compared to 15–24 years, as shown by other authors [64]. On the contrary, Kalichman et al. showed in 2011 that a younger age was associated with anal intercourse [15]. Oral sex is becoming popular among adolescents [65], therefore, there is clearly a need to inform young people on the risks associated with oral and anal sex. Over 60% of the population is under 20 years in the DRC [66] and information on some sexual practices are among Congolese young people’s sexual concerns [67,68]. Far from this need, a 2010 WHO review of behavioral interventions in middle and lower-income countries for HIV positive prevention found that none of the interventions focused on young people [14].

We found that being in a monogamous marriage was inversely associated with oral sex. For anal sex we did not find a significant association. There is scarce published evidence on these associations. Kalichman et al. found that anal intercourse was associated with being unmarried [69]. On the contrary, a Nigerian study among 15–24 year olds showed that anal sex was most often reported in spousal relationships [10]. In any case, we have observed that it is not the civil status alone but the sexual risk behaviors and type of sexual partners which are associated with practicing oral and anal sex.

When information sources, knowledge and perceptions were evaluated, we found that oral sex was associated with a daily use of the Internet and mobile phone. This could be explained by the fact that daily users are more likely to visit pornographic sites, where these kind of sexual practices are common [12,70]. Based on the high use of the mobile phone in this population from Kinshasa (93%), these media are an opportunity to send preventive messages, including those concerning oral and anal sex.

The majority of the surveyed participants thought that the risk of HIV in their community was low and perceiving a low risk was strongly associated with practicing oral sex. Misconceptions regarding HIV risk, transmission and prevention are still common in RDC and may determine sexual risk behaviors [71]. Clear and correct information on the risks associated with each sexual behavior is needed.

As other studies have shown [13,38,40,44,53,59,72], we found that practicing oral or anal sex were associated with other sexual risk behaviors, such as a sexual debut before age 18, having multiple partners, inconsistent condom use or paid sex. Among our young study participants 41% reported having had first sex before 18 years and 10% before 15 (lower rates than shown for Kinshasa in the national health survey and in another recent Congolese study [51,73]). This makes it easier to engage in a greater variety of sexual practices. In fact, a study carried out in Tanzania showed that school students having practiced oral sex and anal sex were more likely to report ever having intercourse [74]. Literature shows that the `normal´ sequence of the sexual experience is to begin with oral sex, continue with vaginal sex and then anal sex, with this last practice being associated with having multiple sexual partners [65,75]. People are more conscious that multiple partnership is a risk behavior for HIV but think that oral sex is, on the contrary, a lower risk practice for HIV transmission than vaginal intercourse and thus, they engage in multiple partnerships not thinking in many other STIs. Also, participants reporting oral or anal sex were more likely to report an inconsistent condom use, as it has been described in other studies [10,76].

Finally, oral and anal sex were associated with reporting having ever been forced to have unwanted sex or unwanted practices, as shown by other authors [50,76,77]. In fact, forced sex itself can include these sexual practices [78,79]. It is important to highlight that HIV/STI transmission risk is even higher in forced sexual practices due to the trauma they can cause [80].

The present study has some limitations. First, we have evaluated sexual behaviors that are perceived as socially unacceptable [20,47,81] and misclassification bias cannot completely be ruled out. However, professional interviewers, individual rooms and anonymity were present to reduce this bias. In any case, this possible misclassification (non-differential) would have biased results towards the null and we still found significant associations. Secondly, we limited questions regarding oral and anal sex to their lifetime prevalence and we did not ask about specific behaviors within each practice, such as the use of condom in oral/anal sex, whether they had these sexual practices with multiple partners or the type of partners involved. Finally, we carried out a cross-sectional analysis of the cohort baseline data and therefore we cannot be sure of the real sequence of the associations found.

Despite these limitations, our study has several strengths. First, this is the first study evaluating the prevalence of oral and anal sex in a Congolese heterosexual population, which contributes to a better knowledge of the HIV epidemic in the country, but also to a better design of the HIV and STI control strategies in SSA. Second, having a high number of participants allowed us to evaluate adjusted associations between many different sociodemographic, knowledge and behavioral factors and extravaginal sexual practices.

### Conclusions

Oral and anal sex are common sexual practices among heterosexual HIV Voluntary Counseling and Testing (VCT) attendees in Kinshasa. They perceive a low HIV risk in the community and have other sexual risk behaviors associated with these extravaginal practices. These practices were commonly not considered as risky despite their strong association with HIV and other STIs. These findings highlight the need of considering oral and anal sex when designing new preventive strategies for heterosexual people in Kinshasa. Information on the risks of these sexual practices needs to be included in preventive campaigns for both men and women, young and adults.
